# Digital adaptation of teaching disaster and deployment medicine under COVID-19 conditions: a comparative evaluation over 5 years

**DOI:** 10.1186/s12909-022-03783-z

**Published:** 2022-10-12

**Authors:** SM Henze, F Fellmer, S Wittenberg, S Höppner, S Märdian, C Willy, DA Back

**Affiliations:** 1 Clinic for Traumatology and Orthopedics, Military Academic Hospital Berlin, Scharnhorststrasse 13, 10115 Berlin, Germany; 2 Center for Musculoskeletal Surgery, Charité - Universitätsmedizin Berlin, Corporate Member of Freie Universität Berlin and Humboldt-Universität zu Berlin, Augustenburger Platz 1, 13353 Berlin, Germany; 3 Clinic for Anesthesiology, Intensive Care, Emergency Medicine and Rescue Service, Military Academic Hospital Berlin, Scharnhorststrasse 13, 10115 Berlin, Germany; 4 Dieter Scheffner Center for Medical Education and Educational Research, Charité - Universitätsmedizin Berlin, Corporate Member of Freie Universität Berlin and Humboldt-Universität zu Berlin, Charité Platz 1, 10117 Berlin, Germany

**Keywords:** Disaster medicine, COVID-19 pandemic, Deployment medicine, Digital education, Undergraduate students

## Abstract

**Background:**

The COVID-19 pandemic has not only brought many aspects of disaster medicine into everyday awareness but also led to a massive change in medical teaching due to the necessity of contact restrictions. This study aimed to evaluate student acceptance of a curricular elective module on disaster and deployment medicine over a 5-year period and to present content adjustments due to COVID-19 restrictions.

**Methods:**

Since 2016, 8 semesters of the curricular elective module took place in face-to-face teaching (pre-COVID-19 group). From the summer semester of 2020 to the summer semester of 2021, 3 semesters took place as online and hybrid courses (mid-COVID-19 group). Student attitudes and knowledge gains were measured using pretests, posttests, and final evaluations. These data were statistically compared across years, and new forms of teaching under COVID-19 conditions were examined in more detail.

**Results:**

A total of 189 students participated in the module from the summer semester of 2016 through the summer semester of 2021 (pre-COVID-19: *n* = 138; mid-COVID-19: *n* = 51). There was a high level of satisfaction with the module across all semesters, with no significant differences between the groups. There was also no significant difference between the two cohorts in terms of knowledge gain, which was always significant (*p* < 0.05). COVID-19 adaptations included online seminars using Microsoft Teams or Zoom, the interactive live-streaming of practical training components, and digital simulation games.

**Conclusion:**

The high level of satisfaction and knowledge gained during the module did not change even under a digital redesign of the content offered. The curricular elective module was consistently evaluated positively by the students, and the adaptation to online teaching was well accepted. Experiences with digital forms of teaching should also be used after the COVID-19 pandemic to create digitally supported blended learning concepts in the field of deployment and disaster medicine and thus further promote the expansion of teaching in this important medical field.

## Introduction

In 2020, the SARS-CoV-2 global pandemic (COVID-19) severely tested the global medical infrastructure [1]. Many health care systems and their personnel worldwide were only partially prepared for such an event, which also required supraregional strategies from the field of disaster and deployment medicine (DDM) [2]. Physicians had to deal with mass casualties of critically ill patients and even had to apply the principles of triage, which had immediate consequences for patients' lives [3]. At the same time, established organizational processes had to be adapted on a large scale in a period of short time, including the reduction of elective surgical procedures and the release of staff for other tasks [1]. Bottlenecks in the care of medical non-COVID emergencies were also described during the pandemic situation [4].

The current impressions emphasized the need, which was already known from previous experience with natural disasters (e.g., hurricanes, earthquakes) or man-made emergencies (e.g., terrorist attacks), for medical personnel to be comprehensively prepared and trained for such extreme situations as well [5]. This need applies not only to the clinical training of residents and specialists but also to medical students. Several papers have described heterogeneous DDM undergraduate course concepts in different countries and have pointed out that students feel inadequately prepared with regard to deployment and disaster medicine and want more DDM learning content within the curriculum [6, 7].

In the context of the COVID-19 pandemic, the necessary contact restrictions also led internationally to a change in the medical teaching and practical training of students [8]. For example, in March 2020, the American Association of Medical Colleges (AAMC) recommended that medical students in the United States be removed from direct clinical contact with patients, thereby eliminating a significant amount of hands-on training [8]. Various studies have shown that online lectures and seminars have become a common online teaching option [9–11]. However, more innovative alternatives, especially for practical learning content, have been less frequently described [12]. In this context, a Libyan survey of 3,348 human medicine students revealed their experiences with first online teaching approaches and their concern that the implementation of online teaching was too scarce and lacking in practical aspects to be adequate enough to count as clinical experiences [13]. This example highlights the importance of diverse implementation and expansion of digital and blended learning approaches, given that the COVID-19 pandemic is still ongoing [8] and furthermore, that important lessons can be learned for the design of teaching in medical faculties [9].

Against this background, the current work was intended to evaluate student acceptance and knowledge gains through a curricular elective module "Deployment and Disaster Medicine" over 5 years. At the same time, a comparison of the course data before and during the COVID-19 pandemic was performed to provide empirical values on how digital components could be integrated into DDM course concepts.

## Material and methodology

### Setting and participants

The curricular elective module "Emergency and Disaster Medicine" (WPM EKM) has been offered to medical students at the Charité—Universitätsmedizin Berlin since the summer semester 2016 on the initiative of the Bundeswehrkrankenhaus Berlin in cooperation with the Charité—Universitätsmedizin Berlin and other civilian stakeholders. The offer of this course in the 6th semester at the end of the 3^rd^ year of undergraduate studies was thereby last arranged for up to 18 students, with 60 teaching hours distributed over a total of 3 weeks.

Within the framework of this course, which is normally held as face-to-face teaching in person, the participants were introduced to aspects of medical care during disasters or crises both at home and abroad. The exact structure and background of the course have been described in a previous publication [14] (during the course of time, only the general amount of weeks and hours was adapted by the medical school for those elective modules from 4 to 3 weeks and from 72 to 60 teaching hours), and an example of the latest schedule adapted to the COVID-19 pandemic regulations is given in Table 1. On a voluntary basis, a multiple-choice knowledge test was administered at the beginning and end of each module (see below), and an online evaluation was conducted by the faculty at the end of the module (see below). Only students enrolled in the course were assessed for data analysis presented here, and there was no control group. “Participation in the course and either the voluntary evaluation and /or both tests was also the only inclusion criterion for this study.”

**Table 1:** Disciplines/topics, module contents and teaching formats in the schedule adapted to the COVID-19 pandemic regulations (m = military teaching staff/ c = civilian teaching staff)

Disciplines/ Topics (staff)	Content (focal)	Teaching format	Teaching Hours (TH)	TH in %
Surgery and Traumatology (c/m)	Gunshot & shrapnel injuries, blast injuries, damage control surgery (DCS), emergency algorithms (ABCDE). Demonstration: Thorax drainage & coniotomy, trauma room training	Webinar, interactive life-streaming	450	16.7
Anaesthesia and critical care (m)	Triage principles & historical development, analgesia under limited conditions, damage control resuscitation (DCR)	Webinar	90	3.3
Emergency medicine (m)	Trauma room management training, prehospital disaster response and incident command systems. Skill training: ABCDE scheme, Stifneck device and pelvic sling, airway management	Webinar, interactive life-streaming	630	23.3
Neurology (m)	Diagnostics and treatment of traumatic brain injuries with limited resources (deployment, austere environments)	Webinar	45	1.7
Radiology (m)	Imaging diagnostics under limited conditions (deployment, austere environments)	Webinar	90	3.3
Psychiatrics, Psychology (c/m)	Post-traumatic stress disorder: backgrounds, diagnostics & therapeutic options, stress management, real patient interviews	Webinar, live Tele-Interview	360	13.3
Disaster medicine (c/m)	Local and regional level disaster preparedness, disaster response with special focus on triage and patient management in MASCAL; organizational structures, esp. emergency medical teams, working conditions and patient collectives in operations; humanitarian aid experience reports. Real time exercise: prehospital and hospital disaster response and preparedness, triage case discussions	Webinar, online live simulation, serious game, online quiz	540	20
Paediatrics (c)	Paediatrics with limited resources	Webinar	90	3.3
Internal medicine, epidemiology and disease control (m)	Outbreak relevant infectious diseases, prevention and treatment (using the examples of West Africa Ebola outbreak and actual COVID-19 pandemic), barrier nursing, nutritional challenges	Webinar	225	8.3
Ethics (m)	Discussion of critical situations in the context of disaster/deployment medicine (e.g., triage)	Group Work	45	1.7
Others/organizational	Welcoming, organizational matters, pre-/posttest, course evaluation	Webinar	135	5

All data were anonymized, and the evaluations complied with the requirements of the Declaration of Helsinki. The responsible ethical committee of the medical school gave its approval (No. EA2/152/22).

### Adjustments to COVID-19 conditions

After 8 semesters under regular face-to-face teaching conditions (pre-COVID-19 group), no face-to-face teaching has taken place since the summer semester of 2020 as part of the COVID-19 pandemic; teaching has largely been conducted digitally (mid-COVID-19 group). The new online teaching approaches include the following:

- **Online seminars:** Classroom seminars were now fully digitally imaged. The Microsoft Teams program (Microsoft Corp., Redmond, WA, USA) and Zoom (Zoom Video Communications Inc., San Jose, Costa Rica) were used for this purpose across the entire faculty. In addition, communication between students and between students and lecturers has been intensified through e-mail and mobile telephony.
- **(Inter)national lecturers:** Some online teaching units were conducted by lecturers living all over Germany, as well as by members of ongoing humanitarian missions abroad.
- **Interactive streaming:** Some practical exercises were filmed live with several cameras so that some practical parts of the course could be demonstrated by the lecturers and observed by the students from multiple perspectives. This approach enabled a concrete approach and detailed filming in direct response to student questions.
- **Online simulations:** Individual teaching units had been replaced by digital planning games and group games with serious gaming characters. The focus was on the team experience of communication and decision-making structures in the event of a disaster (e.g., in the event of a mass casualty incident in a small hospital with a triage situation). One of the used triage games was individually created for this course using www.iseehospital.com (ISEE, Wemmel, Belgium). Another serious game for a triage of a mass casualty event in a small rural remote hospital had been developed within another research collaboration project and is currently under scientific analysis (further details can be asked from the corresponding author of this article).
- **Digital knowledge tests:** The voluntary tests were now digitized (www.surveymonkey.com, Momentive Inc., San Mateo, California, US) and thus scored.

### Pre- and posttests

The pretest at the beginning and the posttest at the end of the curricular elective module each contained 25 multiple-choice questions on DDM, such that the students could earn a maximum of 25 points. The tests represented knowledge tests on the topics to be covered within the course. A validation of all multiple-choice questions had been performed in the beginning of the module by a group of clinical experts in the field of DDM and medical students. Participation in the tests had no impact on passing the module and was voluntary. The participating students were informed in advance with the module study book sent around via email that tests would be performed without grades or any consequence for passing the module. The voluntary participation was communicated orally at the beginning of the module since the tests were always just meant as knowledge determination and not to form any relevant grading for the students. Due to the pandemic, these evaluations were administered as online-only tests beginning in the Summer Semester of 2020. The data were anonymized.

### Student evaluations

After completion of the curricular elective module, voluntary online evaluations were conducted by the participating students. These took place anonymously using standardized questionnaires administered by the faculty. The 13 questions include a five-point Likert scale (corresponding to the question with "strongly agree" (1) to "strongly disagree" (5); "very satisfied" (1) to "not at all satisfied" (5); "very high" (1) to "very low" (5)). They were thematically divided into the evaluation of the general conditions, the teachers, the own learning performance and the learning success. During the semesters, additional individual questions are either added to or removed from the questionnaire depending on the needs of the faculty. These questions were not included in the present evaluation, except for questions in the context of COVID-19, where technical difficulties had been explicitly identified and evaluated since the summer semester of 2020. There was also an option for the participant to provide additional responses and comments for certain questions. Thus, the survey asked which aspects of the course were particularly successful in the context of the students´ perceived organization and which could be improved.

### Data evaluation and statistics

The available datasets were initially accumulated in Excel (Microsoft Corporation, Redmond, WA, USA) and processed accordingly. For the comparative analysis in the context of this study of knowledge gain and acceptance, the semester data from the pre-COVID-19 semesters and the mid-COVID-19 semesters were combined. Statistical analysis was performed using the SPSS program (SPSS Statistics 24, IBM Corp., Armonk, NY, USA) and Student's t test. The free text answers of the evaluation were first pooled and then analyzed independently by two of the authors for recurring statements with qualitative content analysis, according to Mayring, with the inductive category development method [15]. Due to the anonymity of the survey, it was not possible to connect the results of the evaluation to the respective knowledge tests of the students. Ratio data were examined for normal distribution by the Shapiro–Wilk test. To test for statistical significance, ordinal data were analyzed using the Mann–Whitney test and ratio data using the unpaired t-test or Mann–Whitney test depending on normal distribution. Continuous variables were further detailed by mean and standard deviation (SD) and tested for difference.

## Results

In the observed period, 189 students were available for inclusion (pre-COVID-19: *n* = 138; and *n* = 51 mid-COVID). Since the evaluations, as well as the pre- and posttests, were analysed anonymously, no distinction was made between genders. The return rate of the knowledge tests of both the pretests and the posttests was 93% (*n* = 176/189; pre-COVID *n* = 134/138, 97.1%; mid-COVID *n* = 42/51, 82.6%) due to the integration in the lessons. The overall return rate of the evaluation questionnaires was 62.9% (*n* = 119/189; pre-COVID *n* = 92/138, 66.6%; mid-COVID *n* = 27/51, 52.9%).

### Analysis of the evaluations

**Fig. 1:**
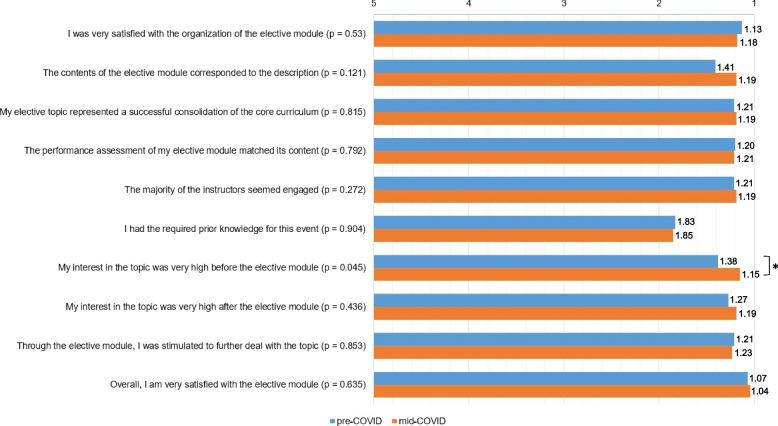
Students´ ratings of evaluation items of the elective module in comparison of the pre-COVID group (*n* = 92) and mid-COVID group (*n* = 27) (with “1” noting the maximum agreement with a statement) (* *p* = 0.045)

All evaluating participants of the course were consistently satisfied with the overall concept of the module (*n* = 119, M = 1.05, SD = 0.02). There were no statistical differences (*p* = 0.635) between students pre-COVID (mean pre-COVID (M_pre_) = 1.07, SD = 0.29, *n* = 92) and those mid-COVID (mean mid-COVID (M_mid_) = 1.04, SD = 0.19, *n* = 27). Most students stated that they had had the necessary prior knowledge for the course, but the students were consistently satisfied with the module as a consolidation of the core curriculum (M_pre_ = 1.21, SD = 0.50, M_mid_ = 1.19, SD = 0.48). There was no significant difference between the pre-COVID group and the mid-COVID group other than the interest for the topic before the module was higher (M_pre_ = 1.38, SD = 0.53, M_mid_ = 1.15, SD = 0.45, *p* = 0.045) in the mid-COVID group (Fig. 1).

Three answers asked about balanced conditions, i.e., if the amount of learning content was too much/not enough in the given time (M_pre_ = 2.80, SD = 0.58, M_mid_ = 3.07, SD = 0.38, *p* = 0.024), if the content was too complex/too simple (M_pre_ = 3.07, SD = 0.36, mean mid-COVID = 3.00, SD = 0.55, *p* = 0.463), and if the individual workload was very high/very low (M_pre_ = 3.34, SD = 0.76, M_mid_ = 3.15, SD = 0.90, *p* = 0.266).

In the text responses, students asked for more time for some of the lessons. Students were willing to invest more time in the module to deepen their knowledge. Especially for the mid-COVID semester students, the structuring of online teaching (e.g., by early information transfer to the students about how the online seminars and digital online games work) and the successful connection between theory and practical simulation were emphasized and praised. In some cases, however, the students also indicated difficulties when attending and watching the live online seminars of the module on their mobile devices. Nevertheless, the students in the mid-COVID group also indicated that they were very satisfied with the organization of the module (M_mid_ = 1.18, SD = 0.48). This was similarly evident in the pre-COVID group (M_pre_ = 1.13, SD = 0.36) but not statistically significant (*p* = 0.53).

### Results of the pre- and post-knowledge tests

**Fig. 2:**
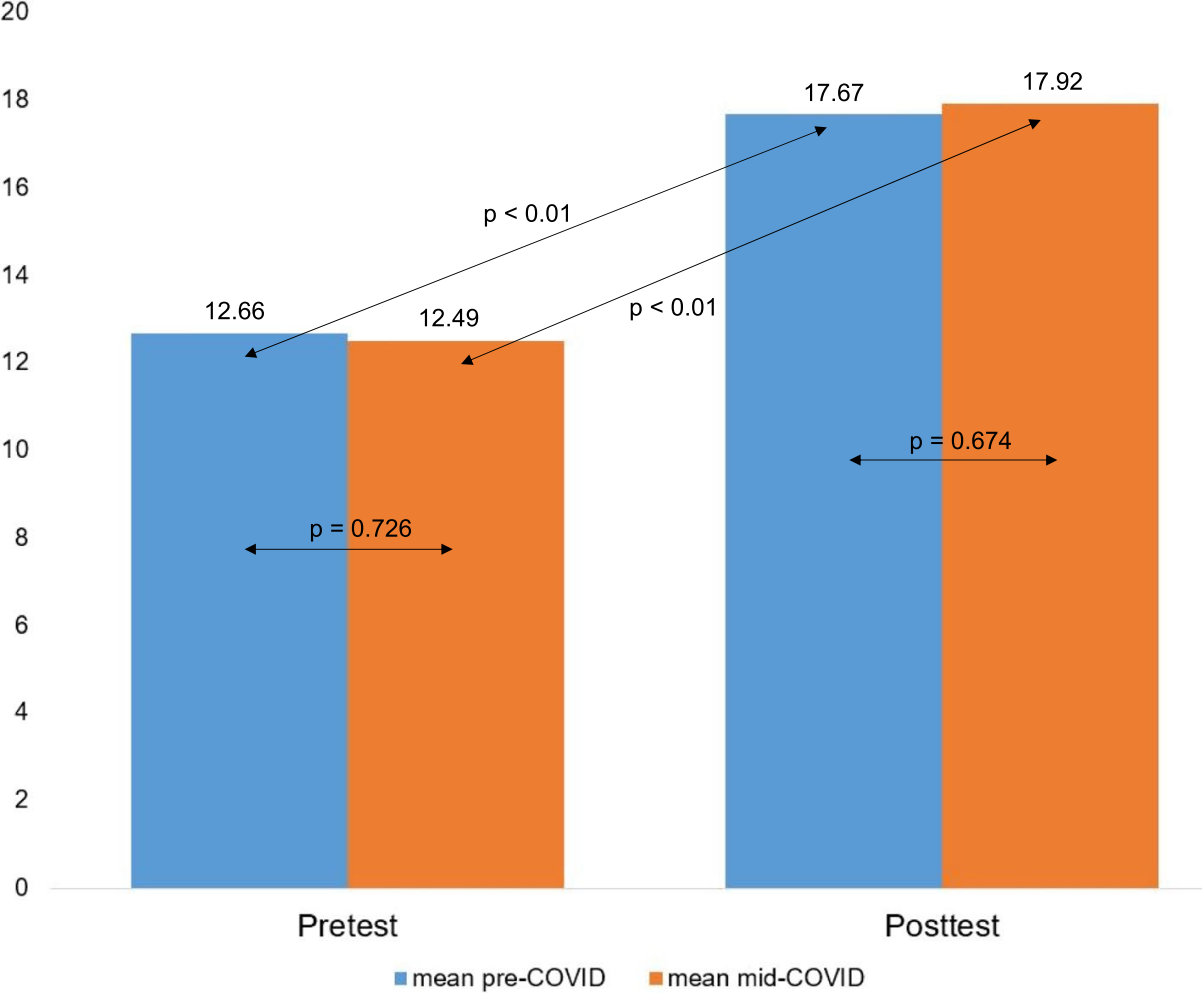
Comparison of the pre- and post-knowledge test results of the pre-COVID group (n_pre_ = 134) and the mid-COVID group (n_mid_ = 42) with no significant difference between pretests (*p* = 0.726) and posttests (*p* = 0.674) of both groups in direct comparison. Statistically significant gain in knowledge from pretest to posttest in both pre-COVID and mid-COVID groups (*p* < 0.01)

In the overall evaluation among the voluntary tests participants of all semesters (*n* = 189, n_pre_ = 134, n_mid_ = 42), there was a mean of 12.57 points (SD = 2.59) per semester in the pretests, with no significant differences (*p* = 0.726) between the pre-COVID (M_pre_ = 12.66, SD = 2.56) and mid-COVID groups (M_mid_ = 12.49, SD = 2.69). The overall analysis of the posttests showed a mean score of 17.73 points (SD = 2.53) without any significant difference between the pre-COVID and mid-COVID groups (M_pre_ = 17.69, SD = 2.47 / M_mid_ = 17.88, SD = 2.73) (*p* = 0.674). For all semesters, a significant gain in knowledge between the initial pretest and the final posttest could be noted (*p* < 0.01) (Fig. 2).

### Subjective experiences with COVID-19 module adaptation

Based on the well-established communication structures between the approximately 20 different lecturers and clinical teachers of the module, there were only a few problems with convincing members of the faculty to offer the existing content in a remote and digital way. A few of the lecturers who were not used to online teaching tools were hesitant in the beginning. However, this changed quickly after their first experiences with online teaching, which were conducted under close guidance by the organizational staff.

Seminars and presentations had only been adapted slightly to online teaching and met with students who were already well prepared to join this kind of theoretical lesson approach from the summer semester of 2020 onwards.

More complex was the preparation of the live streaming of the practical trainings due to the use of different cameras (professional, mobile phone or laptop) or channels, since just one video could be transmitted via the universities’ MS Teams, but different camera-perspectives were intended to be offered. This issue was solved by using different accounts (Microsoft Teams, Zoom) with different live streams, thus enabling the students to switch viewing angles on their own. Additionally, the performance of the streaming required some flexibility, as no professional video team was available. The performance of the serious games and virtual role plays was rather easy, since digital versions of these teaching contents had already existed previously.

The complexity and importance of clear communication pathways was made visible by a serious game during which students had to communicate via MS Teams, Zoom, e-Mail and mobile phones in parallel. As a positive aspect, these kinds of offers also enabled teamworking structures and the active involvement of all remote students. Furthermore, remote teaching staff could be integrated as well, thereby enabling lecture streaming from different cities in Germany, as well as from ongoing missions (humanitarian and military) abroad.

Finally, tests and evaluations were translated without problems into online versions. Overall, due to a high level of positive attitudes and flexibility among the faculty, the necessary adaptations required under COVID-19 restrictions could be well addressed.

Due to the use of pre-existing hardware (e.g. videocall-enabled computer) and software (e.g. MS Teams by the Charité) no additional costs incurred. Other hardware, such as a GoPro camera (GoPro Inc., San Mateo, CA, USA) or different smartphones for live video-transmission were used from own material of the lecturers. Challenges were to find a room within the hospital for everyday mere use for seminars and as practical skill transmission, as well as to establish a technical support from the IT department.

## Discussion

Within this study, it could be shown that even under COVID-19 conditions, a high level of agreement among the participating study participants, as well as significant knowledge gains, could be achieved. To make the digital transfer of knowledge attractive, various new teaching formats were introduced. To the authors' knowledge, these outcomes are described herein for the first time with regard to teaching in the field of deployment and disaster medicine (DDM).

The results of the study confirm that the DDM module has been very well received by students since 2016. The biggest limitation of the mid-COVID-19 course design seems to be the limited physical hands-on experience in clinical and emergency surgical skills labs, as described in the literature [13]. This limitation may have resulted in an increased perceived complexity of the course and possibly, even though this suggestion was not tested, a decrease in practical clinical surgical proficiency.

All other analysed parameters, however, showed an equivalent or even better long-term course result. Fittingly, the subjective and objective knowledge increase in the mid-COVID-19 group was comparably high with that in the pre-COVID-19 group, and the interest in the topic before the module was even increased. Likewise, the study participants attested that they felt they had achieved a great learning success and saw themselves better prepared after completion of the module. Other authors have also confirmed such observations for pre-COVID-19 courses in this subject area [16]. Although these were only subjective statements, they are interesting in the COVID-19 context since in other publications, medical students have stated that they sometimes see themselves as less prepared for clinical practice due to restrictions [8, 13].

In the present evaluation, the students also considered DDM to be instructive study content. Its importance is supported by international authors who also support maximizing the workforce in case of disaster or a health emergency [17]. The findings with an overall lower response rate in the mid-COVID group compared to the pre-COVID group could be explained by the fact that evaluation during COVID was conducted after completion of the module, maybe resulting in a decreased motivation of filling in the document.

In general, DDM is an important subject area that has thus far received only very variable attention in medical education internationally. In addition to a few national approaches, some of which already included blended learning components [18], smaller initiatives at individual medical schools have been described, with varying content and scope [19–22]. Most of these were single courses [20, 21] or sequences of courses that range over a few semesters [19, 22]. This may be because these courses are often added to regular curricular teaching, which requires a certain resilience on the part of the organizing teachers. Therefore, it seems important to analyse longer-term experiences scientifically, as seen in the present work.

Adapting teaching to predominantly digital offerings has been an international challenge [11, 23, 24]. Thus far, different solutions have been found. For example, webinars as a substitute for seminars, video streams of prerecorded lectures, and digital clinical skills labs as part of blended learning concepts have been introduced across universities [10]. In addition, telemedicine has been massively expanded [25]. These changes have enabled students in the USA, for example, to continue to have contact with real patients within the framework of interactive digital visitations and video consultations. Corresponding telemedicine course offerings for continuing education have been well received and implemented by students [26]. Furthermore, immersive techniques such as virtual reality or augmented reality solutions have been increasingly applied [27]. These solutions have been supplemented by innovative concepts such as serious games and are not only limited to studies but also find their way into professional everyday life [12, 28]. Analogous to the experiences described herein, a study of British students showed that they perceived a live connection to practical instruction to be extremely effective, presumably because of the possible discussion and direct communicative interaction with the lecturers [9].

In perspective, digital forms of teaching can not only offer students the flexibility to learn at their own pace but also prepare them for the possible use of digital techniques in clinical practice [9, 29]. Moreover, for online teaching, forms of implementation matters. A survey in the UK, for example, highlighted limitations due to family distractions, problems with internet connectivity, and the timing of teaching sessions [9].

From a staff perspective, impressions gained by the authors showed that with a high level of positive attitude and flexibility among the teaching staff, the necessary adaptations under COVID-19 could be well addressed in this case, which goes along with other published opinions [30].

A limiting factor is that the level of voluntary participation and evaluation in the current study represents a positive selection of students who were already interested in the topic. However, obligatory participation in the course format for all students during a semester could show other results. In addition, it must be noted that with 189 students, only a small sample of the students enrolled at the university in the respective semesters participated in the curricular elective module. A further limiting factor was the lack of a control group for the pre-post-tests of the participants analyzed here, making this an uncontrolled study. This should be repeated in future studies on this topic. Another drawback of this study was the lack of collecting demographic data like gender or age of the participants. This was due to the retrospective character of the here presented analysis and the fact that also the medical schools´ own evaluations did not include these demographic data, since students were regularly enrolled the 6^th^ semester. The fact that the intervention achieved a significant knowledge gain through the course was to be expected. However, future evidence of the sustainability of knowledge gain among students, even over a longer period after completion of the module, should also be evaluated. This could be done by re-evaluating the level of knowledge and medical skills after graduation and comparing the results with those of other graduates who did not participate in the here described course format. Since a reliability analysis of the tests has not been done so far, this should be addressed in future studies.

As an outlook into the future, it can be postulated that the COVID-19 pandemic has shown that a shift towards online teaching is also possible in the field of deployment and disaster medicine. Certain practical content, such as emergency surgical techniques, will not be able to be replaced digitally in the near future. Thus, the highest potential can be seen in blended learning concepts.

Since sophisticated online teaching media are often time-consuming to create [31], scientifically supported development should begin promptly. In particular, the possibilities of virtual reality, augmented reality or serious games should be the focus [27, 32, 33]. Already developed pan-national concepts for disaster medicine education of students at medical faculties should also be extended and elaborated with regard to the COVID-19 pandemic [34].

## Conclusion

The COVID-19 pandemic has emphasized how elementary and relevant principles of deployment and disaster medicine (DDM) are for the comprehensive education of medical students. The data from a curricular elective module analysed herein show that, on the one hand, offered DDM teaching courses can be well integrated and maintained at a university level even in the medium term. By using different online teaching media, a good satisfaction level of the students with the teaching offered, as well as a significant knowledge gain, can be achieved even under COVID-19 contact restrictions, which is analogous to pre-COVID-19 conditions. Based on the experience gained, the strengths of online teaching should be further strengthened, with the addition of hands-on teaching, to make education in DDM even more effective and interesting through blended learning concepts.

## Data Availability

The datasets generated and analysed during the current study are available from the corresponding author on reasonable request.
